# Genetics of Type III Bartter Syndrome in Spain, Proposed Diagnostic Algorithm

**DOI:** 10.1371/journal.pone.0074673

**Published:** 2013-09-18

**Authors:** Alejandro García Castaño, Gustavo Pérez de Nanclares, Leire Madariaga, Mireia Aguirre, Alvaro Madrid, Inmaculada Nadal, Mercedes Navarro, Elena Lucas, Julia Fijo, Mar Espino, Zilac Espitaletta, Luis Castaño, Gema Ariceta, RenalTube Group

**Affiliations:** 1 Research Unit, Ciberer, Cruces University Hospital, Bizkaia, Spain; 2 Pediatric Nephrology, Cruces University Hospital, Bizkaia, Spain; 3 Department of Pediatrics, School of Medicine and Odontology, University of Basque Country UPV/EHU, Bizkaia, Spain; 4 Pediatric Nephrology, Materno Infantil Vall d’Hebron Hospital, Barcelona, Spain; 5 Pediatric Nephrology, Virgen del Camino Hospital, Pamplona, Spain; 6 Pediatric Nephrology, La Paz University Hospital, Madrid, Spain; 7 Pediatrics, Manises Hospital, Valencia, Spain; 8 Pediatric Nephrology, Virgen del Rocío Hospital, Sevilla, Spain; 9 Pediatric Nephrology, Fundación Alcorcón University Hospital, Madrid, Spain; 10 San Ignacio University Hospital, Bogotá, Colombia; 11 Pediatric Nephrology, Asturias Central University Hospital, Oviedo, Asturias, Spain; 12 Pediatric Nephrology, Nuestra Señora de Candelaria University Hospital, Tenerife, Canarias, Spain; University of Bonn, Institut of experimental hematology and transfusion medicine, Germany

## Abstract

The p.Ala204Thr mutation (exon 7) of the *CLCNKB* gene is a "founder" mutation that causes most of type III Bartter syndrome cases in Spain. We performed genetic analysis of the *CLCNKB* gene, which encodes for the chloride channel protein ClC-Kb, in a cohort of 26 affected patients from 23 families. The diagnostic algorithm was: first, detection of the p.Ala204Thr mutation; second, detecting large deletions or duplications by Multiplex Ligation-dependent Probe Amplification and Quantitative Multiplex PCR of Short Fluorescent Fragments; and third, sequencing of the coding and flanking regions of the whole *CLCNKB* gene. In our genetic diagnosis, 20 families presented with the p.Ala204Thr mutation. Of those, 15 patients (15 families) were homozygous (57.7% of overall patients). Another 8 patients (5 families) were compound heterozygous for the founder mutation together with a second one. Thus, 3 patients (2 siblings) presented with the c. -19-?_2053+? del deletion (comprising the entire gene); one patient carried the p.Val170Met mutation (exon 6); and 4 patients (3 siblings) presented with the novel p.Glu442Gly mutation (exon 14). On the other hand, another two patients carried two novel mutations in compound heterozygosis: one presented the p.Ile398_Thr401del mutation (exon 12) associated with the c. -19-?_2053+? del deletion, and the other one carried the c.1756+1G>A splice-site mutation (exon 16) as well as the already described p.Ala210Val change (exon 7). One case turned out to be negative in our genetic screening. In addition, 51 relatives were found to be heterozygous carriers of the described *CLCNKB* mutations. In conclusion, different mutations cause type III Bartter syndrome in Spain. The high prevalence of the p.Ala204Thr in Spanish families thus justifies an initial screen for this mutation. However, should it not be detected further investigation of the *CLCNKB* gene is warranted in clinically diagnosed families.

## Introduction

Bartter syndrome (BS), which was first described in 1962 [1], is a heterogenic autosomal recessive disorder of the salt reabsorption at the thick ascending limb (TAL) of Henle’s loop. BS includes a group of closely related hereditary tubulopathies characterized by hypokalemia, metabolic alkalosis, hyperreninemia, hyperaldosteronism with normal or low blood pressure, renal salt loss and hyperplasia of juxtaglomerular apparatus [2]. Two phenotypic variants have been described in patients with BS, one presenting early in life with severe salt loss and marked prostaglandinuria, called “neonatal BS”, and another one with a milder phenotype or later onset of the disease, named “classic BS” [3-5]. Furthermore, patients with mutations in the same gene can present different phenotypes [6].

Loss-of-function mutations of several genes encoding those transporters involved in salt reabsorption at the TAL cause different types of BS: *SLC12A1* (Na-K-2Cl cotransporter NKCC2) causes type I BS (OMIM #601678) [7], *KCNJ1* (K+ channel, ROMK) causes type II BS (OMIM #241200) [8] and *CLCNKB* (Chloride Channel Protein ClC-Kb) causes type III BS (OMIM #607364) [9]. Loss-of-function mutations in the *BSND* gene, that encodes barttin, an essential beta subunit for ClC chloride channels, cause type IV-A BS with sensorineural deafness (OMIM #602522) [10]. On the other hand, gain-of-function mutations in the *CASR* gene (OMIM +601199) cause type V BS [11]. Finally, simultaneous mutations in both the *CLCNKB* and *CLCNKA* genes cause type IV-B BS (OMIM #613090) [12].

As mentioned above, type III BS is caused by mutations in the *CLCNKB* gene (OMIM *602023), on chromosome 1p36. It spans 20 exons, 19 encoding for the chloride channel protein ClC-Kb [13]. Three splice transcript variants have been found for this gene. ClC-Kb is associated with another chloride channel, ClC-Ka, which is encoded by the *CLCNKA* gene (OMIM *602024). *CLCNKB* and *CLCNKA* genes are separated by approximately 10 kb of genomic sequence, and they have 94% of sequence identity.

The ClC-Kb protein, which is expressed predominantly in the TAL, distal tubule and collecting duct, is required to ensure the chloride (Cl^-^) exit on the basolateral cellular side [14]. Thus, impaired ClC-Kb function reduces Cl^-^ exit and decreases Na-K-Cl reabsorption through the Na-K-2Cl cotransporter (NKCC2) [7-9] by modifying the transepithelial voltage gradient and causing salt loss in urine [15].

The ClC-Kb is formed by 687 amino acids and is present as a homodimer at the membrane. Each subunit is composed of 18 α-helices (A-R), with 12 transmembrane domains and intracellular amino and carboxyl termini, the latter containing two cystathionine-β-synthase (CBS) domains [16].

At present, according to the Human Gene Mutation Database (HGMD database, Biobase-International, www.hgmd.cf.ac.uk), 75 mutations have been described in the *CLCNKB* gene, including gross deletions (8), small deletions (6), small insertions (4), complex rearrangements (2), nonsense or missense mutations (45) and splice site mutations (10).

The genetic diagnosis of type III BS is complex because of its large phenotypic variability. Indeed, mutations in the *CLCNKB* gene may cause phenotypes that overlap with other types of BS and Gitelman syndrome (OMIM #263800) as well [6,17].

Our group previously described a founder mutation in the *CLCNKB* gene (c.610G>A; p.Ala204Thr) that causes type III BS in Spain [18]. To date, our experience has been that almost all Spanish patients clinically diagnosed of type III BS present this mutation. However, we have seen an increasing number of patients with a clinical diagnosis of type III BS who do not carry the p.Ala204Thr mutation. Thus, genetic analysis of the entire *CLCNKB* gene is indicated. The aim of this study was to perform a comprehensive genetic analysis in our cohort of patients with type III BS, and to devise a strategy for gene analysis that is appropriate for the Spanish population.

## Methods

### Ethics Statement

The study was approved by the Comité Ético de Investigación Clínica (CEIC) from the Cruces University Hospital. The patients and their relatives provided their written informed consent to participate in this study. In case of the minors included in the cohort, we obtained written informed consent from their guardians prior to enrolment in the study.

### Population

A total of 26 patients (18 females), of mean 3,4 (range: 0,3-25) years of age, belonging to 23 families with type III BS and unaffected available relatives (n=57) were analyzed. 10 patients of our cohort were included in our previous manuscript, in which we described the founder effect of the c.610G>A; p.Ala204Thr mutation in Spain (Table 1) [18]. Clinical diagnosis was performed at different hospitals, based on symptoms and biochemistry disturbances (Table 1). Most patients demonstrated a decreased distal Cl^-^ reabsorption during hypotonic saline diuresis [19]. In 20 out of 26 cases nephrocalcinosis was not detected. One family (SOR051) was known to be consanguineous.

**Table 1:** Clinical and biological characteristics of BS patients.

*Patient*	*Age at diagnosis (years*)	*Gestational Age (weeks*)	*NC*	*PH*	*Body Height (SDS*)	*Body Weight(SDS*)	*pH blood*	*Na* ^*+*^ * mEq/l*	*K* ^*+*^ * mEq/l*	*Cl* ^*-*^ *mEq/l*	*HCO*_*3*_^*-*^ * mEq/l*	*Mg* ^*2+*^ * mg/dl*	*Creati-Nine mg/dl*	*FENa* ^*+*^ ** ^*n*^ *%*	*FEK* ^*+*^ * %*	*FECl* ^*-*^ * %*	*Renin ng/ml/h*	*Aldosterone pg/ml*	*UCa* ^*2+*^ */Cr mg/mg*
*SOR003	6,8	36	-	+	-0,1	-1,3	7,53	134	1,4	100	35	0,9	0,7	1,2	49,9	2,9	20,9	945	0,4
*SOR005	3,0	40	+	+	-1,0	-1,6	7,50	130	2,0	85	28	2,2	0,5	2,8	50,5	4,0	60,5	484	0,5
*SOR008	2,0	40	+	-	-3,2	-4,9	7,47	134	2,0	88	28	1,5	0,7	1,5	62,3	2,1	NA	NA	0,3
*SOR009	2,0	36	-	+	-3,5	-4,5	7,31	143	1,7	100	20	NA	0,7	1,3	76,8	2,4	13,6	NA	0,3
SOR023	1,8	40	-	-	-3,7	-3,2	7,47	140	3,3	85	24	1,9	0,4	1,2	27,5	2,1	53,4	267	0,7
*SOR025	2,3	40	-	-	-2,0	-3,7	7,57	132	2,3	96	27	1,6	0,5	1,2	34,2	2,0	215	5610	0,2
*SOR026	0,7	40	-	+	-1,9	-3,7	7,42	136	3,3	90	37	2,5	0,2	0,1	7,20	0,2	13,6	1654	0,2
SOR039	1,4	41	-	-	-2,3	-4,0	7,49	138	1,6	93	28	2,8	0,3	2,3	54,0	3,5	86,6	1281	0,2
SOR045	0,7	NA	-	+	-2,3	2,0	7,48	133	2,9	79	40	2,3	0,4	0,2	13,4	0,3	NA	670	NA
SOR047	0,7	NA	-	-	-1,9	-3,5	7,65	137	2,5	83	31	3,1	0,3	0,1	17,7	0,3	NA	760	0,1
*SOR048	2,0	42	-	+	-1,2	-2,3	7,38	126	2,7	82	27	3,0	0,2	0,6	14,2	0,6	77,4	282	0,2
SOR050	0,7	41	-	-	-5,3	-5,3	7,50	140	2,2	99	31	2,5	0,3	0,2	20,8	NA	NA	NA	0,0
SOR051	25	NA	+	NA	NA	NA	7,34	134	2,3	98	24	2,6	2,7	NA	NA	NA	NA	NA	NA
SOR062	3,0	40	+	-	-1,9	-2,7	NA	130	1,9	89	31	2,4	0,3	0,9	42,0	1,4	80,4	1608	1,1
SOR073	0,7	40	-	-	-1,0	-3,2	7,47	139	3,3	97	30	2,2	0,4	1,0	25,0	0,3	8,30	1370	0,4
*SOR011	11	NA	-	-	-2,3	NA	7,45	138	2,0	93	29	1,7	0,5	0,8	31,4	1,1	31,1	1015	NA
*SOR024	0,9	NA	-	NA	NA	-5,0	NA	NA	1,8	NA	NA	NA	0,5	NA	NA	NA	NA	NA	NA
*SOR024	17	40	-	-	1,8	1,0	NA	132	2,6	134	33	1,3	0,4	NA	NA	NA	195	114	NA
SOR054	0,3	NA	-	-	-0,3	-2,3	7,51	131	2,3	80	33	2,2	0,5	0,3	42,1	0,6	NA	NA	0,0
SOR054	0,3	NA	-	-	-2,1	-2,2	7,46	134	2,9	86	28	2,2	0,7	0,5	25,0	1,2	21,0	185	0,0
SOR054	0,5	NA	-	-	-0,6	-1,5	7,40	145	2,2	81	26	NA	0,5	0,2	30,9	NA	60,6	220	0,5
SOR057	0,7	36	+	+	-3,5	-6,2	7,62	134	2,3	87	35	2,9	0,3	0,1	24,3	0,5	39,0	473	0,4
SOR064	0,8	41	-	+	-1,7	-2,6	7,55	134	3,1	81	43	2,3	0,3	0,2	8,20	0,2	19,9	1584	0,5
SOR063	0,4	41	-	-	-1,0	-2,9	7,59	133	2,7	94	33	2,3	0,3	0,3	35,2	1,3	30,0	384	0,1
SOR076	1,0	NA	-	-	-1,6	1,5	7,46	138	2,7	NA	28	NA	0,6	NA	NA	NA	50,0	480	NA
SOR021	2,5	NA	NA	NA	NA	NA	NA	NA	NA	NA	NA	NA	NA	NA	NA	NA	NA	NA	NA
**MEAN**	3,4	40			-1,8	-2,7	7,48	135	2,4	91	30	2,2	0,5	0,8	32,9	1,4	59,7	1020	0,3
**SD**	5,7	2			1,4	2,1	0,08	4,37	0,5	11	5	0,6	0,5	0,7	18,2	1,2	58,2	1226	0,3
**MEDIAN**	1,2	40			-1,9	-2,9	7,47	134	2,3	89	29	2,3	0,4	0,6	30,9	1,2	44,5	670	0,3
**MIN.**	0,3	36			-5,3	-6,2	7,31	126	1,4	79	20	0,9	0,2	0,1	7,2	0,2	8,30	114	0,0
**MAX.**	25	42			1,8	2	7,65	145	3,3	134	43	3,1	2,7	2,8	76,8	4	215	5610	1,1

* Patients included in our previous manuscript [18]. Abbreviations: NC, nephrocalcinosis; PH, polyhydramnios; + yes; - no; NA, not available; SDS, standard deviation score in comparison with age- and sex-matched reference population; FE, fractional excretion; U, urinary; Ca^2+^/Cr, calcium/creatinine ratio; SD, standard deviation; MIN, minimum; MAX, maximum.

Biological samples from the patients were received at the Molecular Genetic Laboratory at Cruces University Hospital, Bizkaia, Spain. Most samples were sent from the Pediatric Nephrology Division at Cruces University Hospital (n=14) and Renaltube team (n=7), a network tool for clinical and genetic diagnosis of primary tubulopathies (www.renaltube.com). A few samples (n=5) were received from other hospitals in Spain.

### DNA analysis

Genomic DNA was extracted from peripheral blood leukocytes according to manufacturer’s instructions (QIAamp® DNA Blood Mini Kit, QIAGEN, Germany).

The exon regions and flanking intronic sequences of the *CLCNKB* gene (Ensembl identifiers: gene ENSG00000184908; transcript, ENST00000375679) were screened for mutations by polymerase chain reaction (PCR) followed by direct sequencing.

We used newly designed primers for non-coding exon 1 and promoter region (according to SwitchGear Genomics), and previously described primers [20-22] to amplify exons 2-20 together with their splice sites (Table 2). Due to the high (>90%) sequence similarities between *CLCNKB* and *CLCNKA*, and their close location on chromosome 1, specific primers for individual exons were used only when available, this is, when both genes’ sequence was different enough to specifically amplify the *CLCNKB* gene. For the cases it was not possible to directly amplify the *CLCNKB* gene’s individual exons, we generated by Long-range PCR three large fragments (named FD, FE and FF), specific for the *CLCNKB* gene. Then, we used nested PCR with internal specific primers to analyze exons 4 (within product FD), 8, 9-10 (within product FE) and 13-14 (within product FF) (Table 2). All PCR reactions were performed in a total volume of 50µl, in presence of 10% DMSO (dimethyl sulfoxide). Annealing temperatures and amplicons’ sizes are shown in Table 2.

**Table 2:** PCR and sequencing primers for the analysis of the human *CLCNKB* gene.

Exon/ Promoter	Forward primer (5´- 3´)	Reverse primer (5´- 3´)	Annealing T (°C)	Amplicon Length (bp)
Promoter	* CTCTCCCCATTCACAGGGTG	* **TGGACAGGTGTGTGTTCCAA**	56,3	966
	† CGGCCTCC**GT**G**ATCTTAGA**C	† AGCATGACCACAGCCTCC		
1	* CTCTGTGCAGCT**A**TG**G**TGGG	* CTG**T**C**C**A**C**CT**AT**GAGCACCC	56.3	429
2	‡ ACTGGAAGGGCC**T**AGAGGCAG**T**	‡ GATGTCCTGAGTGGTCC**T**CC**AG**	60	231
3	§ **CACTGTGTCACCACTGTCACC**	§ **AGGAGTAAAGCCAGGACCAGA**	60	460
FD	|| ¶ TGCCCCA**C**C**C**T**GT**GC**C**G**T**GAC	|| ¶ GGGTGG**T**TGGG**A**TGCCCTCAC	66,8	2558
4	|| # GAGGCTGTGGGTGCCTCCCTG	|| # A**G**TGGGGACTGGC**G**T**A**GCGAC	61,4	200
5-7	§ **AGATCTTGTCCCCAAAGGAAA**	§ GGCTGAAGTGAGAAC**T**AGAATGA	60	950
	† AGTGGTATGG**G**CAGG**G**GT	† AGGTAGGCAGCCATCATCAC		
	† CACCTGTCTGT**G**ATGAT**G**G	† GGG**TA**GGGTGG**T**TGGG**A**		
FE	|| ¶ **CCCTCCTGGCCCTGCCCAC**	|| ¶AGCTC**G**CTGAGA**G**GTCCCCAG	66,8	2082
8	|| # **G**G**A**GGGCCCACCTGA**G**ATCAG	|| # GCAGGGCCAGGGTCAGGCAG	61,4	193
9-10	|| # CGCCATCTTGGCTCCCCACTG	|| # AGCTC**G**CTGAGA**G**GTCCCCAG	61,4	374
11-12	§ **CTGACCCCACAGGTTCTGT**	§ ** CCAGGGCAGAGGTTAGAGGC	60	1099
	† **GC**C**T**CCTTTGC**G**TGT**A**T	† TAACCAGGAAGAAGGCAAGG		
	† GGTTCACCATCTTTGGGA	† CTGA**C**CTCCC**GA**AGC**TG**T**A**G		
FF	|| ¶ GTC**G**GGCTCTGG**G**CT**C**A**TG**TC	|| ¶ CAGTCAGCCTGAGGTGGGCAC	66,8	2660
13-14	|| # TCTAGGACACTCCCCTGTCCC	|| # CCCTGGGGAACCACCAGCCAA	61,4	519
15	†† CATCACT**C**CCTCGTGGCTCCTG	†† CT**A**CGGTGG**C**GTTTCTTTTT**C**G	60	518
16	†† **G**C**TA**AAGTG**G**AGCTGGTCTG	†† **GCAACAAGGATTTGGAGG**	60	750
	† CCACAGCATCACCACACT	† CACAACCTTGACCACCTCCT		
17-18	§ **CACAATAGCCCCATAGGAACA**	§ C**T**CTCCCA**C**TTCCCTCATCTC	60	672
	† GGTC**A**GAGAGAGGCATCCTG	† ATGTCCTGGAGACACTGCTG		
19	|| GGGC**A**CC**T**TCTACCCTCCAGTG	|| G**T**CTTCTCAGGCAT**A**GGT**T**CCCTG	60	187
20	|| CTACATCCCCCC**G**CACC**A**CCAC	|| AGGGTCTCAGCCCAACCTC	60	153

According to DNA sequence (Ensembl: ENST00000375679); Bases that differs from the *CLCNKA* gene (Ensembl: ENST00000331433) are marked in bold; * Newly designed primers; † Newly designed internal primers for sequencing; ‡ Previously designed primers [20]; § Previously designed primers [21]; || Primers kindly provided by the Hôpital Européen Georges Pompidou, Service de Génétique, Paris; ¶ Specific primers for long-range PCR; # Internal specific primers for nested PCR; ** Reverse primer modified (last base removed because it is a SNP); †† Previously designed primers [22].

PCR products were then purified by extracting them from agarose gels using QIAquick Gel Extraction Kit (QIAGEN), according to the manufacturer’s specifications.

Purified amplified products were directly sequenced with fluorescent dideoxynucleotides (BigDye Terminator v3.1 Cycle Sequencing Kit, Applied Biosystems, Foster City, CA). We used newly designed internal primers for direct sequencing of fragments 5-7, 11-12, 16, 17-18 and promoter (Table 2). In the rest of exons, the same primers as in PCR were used for direct sequencing.

Excess dye terminators were removed (ethanol-ethylenediaminetetraacetic acid-sodium acetate) and samples were denatured and loaded onto an ABI 3130xl Genetic Analyzer (Applied Biosystems, Foster City, CA). Pathogenic effect of specific DNA variants was assessed using Ensembl (www.ensembl.org, EMBL-EBI) and HGMD database. Missense DNA variants, not listed either in the Ensembl database or in the HGMD database, were assessed using prediction pathogenic software: Mutation t@sting (www.mutationtaster.org), PolyPhen-2 (genetics.bwh.harvard.edu), SIFT (sift.jcvi.org), SNPs & GO (snps-and-go.biocomp.unibo.it) and NetGene2 Server (www.cbs.dtu.dk) for variations in the splice sites. DNA mutations were named according to the Human Genomic Variation Society guidelines (HGVS, www.hgvs.org).

For the purpose of detecting large deletions or duplications a commercially available kit, SALSA MLPA (Multiplex Ligation dependent Probe Amplification) probemix P266-B1 (MRC, Holland, Amsterdam, The Netherlands) was used. This kit contains probes for 14 out of 20 exons of the *CLCNKB* gene and more telomeric probes, including 2 probes for the *CLCNKA* gene, 1 probe for the *CASP9* gene and 1 probe for the *PRDM2* gene.

In order to analyse the remaining exons without a MLPA probe (exons 4, 7, 9, 12, 16 and 20) we performed Quantitative Multiplex PCR of Short Fluorescent Fragments (QMPSF) assays. QMPSF is a simple method based on the simultaneous amplification of short genomic sequences under quantitative conditions, using dye-labelled primers, and on the comparison of profiles generated from tested and control DNA, and is considered a very sensitive method for the detection of genomic deletions and duplications. Briefly, we simultaneously amplified our exons of interest together with exon 7 of the *HNF1β* gene (used as a control fragment), stopping the reaction at the exponential phase of the amplification in order to get a semiquantitative estimation of each PCR product. Fragments were then separated by capillary electrophoresis (ABI 3130xl Genetic Analyzer) and analysed. Primer sequences, annealing temperatures and amplicons’ sizes are shown in Table 3. Two QMPSF assays were performed for each sample to easily discriminate between all amplicons.

**Table 3:** PCR Primers for QMPSF.

**QMPSF1** Exon	Forward primer (5´- 3´)	Reverse primer (5´- 3´)	AnnealingT (°C)	Amplicon Length (bp)
4	*TGTACCCTGTGGCCCTCGTC	*/5’6-FAM/ **C**G**CAGAAGA**TCCT**CC**CACCA	50	323
12	*TCGCTGTTCGACAACCACTC	*/5’6-FAM/**CCACCAGCTGCGATGAGGT**	50	251
16	*/5’6-FAM/GGGTCT **C**ACATCCC**T**GACT**GT**	*CCTTGACCACCTCCTCCAG**T**	50	157
17-18	*/5’6-FAM/CCTCCTTCC **T**GGGCTCC**T**	*GCAGCC**TGCA**GCCAAGAT	50	231
†7 (*HNF1* β gene)	‡/5’6-FAM/**TCAACACCTCCCAAGCACA**	‡**TGAGTCACAGCTGCCATGA**	50	197
**QMPSF2** Exon	Forward primer (5´- 3´)	Reverse primer (5´- 3´)	AnnealingT (°C)	Amplicon Length (bp)
6-7	*/5’6-FAM/CGTGCACCTGTCTGT **G**ATGAT**G**	*GGAGCTGCAAAGACTGTGGC	50	244
9-10	*CAGTTTCCGGGTGGACGTT	*/5’6-FAM/ **A**ACC**T**ATTG**T**TC**C**TGATGAAGC**C**	50	270
20	*/5’6-FAM/GCTCTACTATT **T**A**C**CC**A**G**A**AA**CCAC**	*CTGGCGGATTTGTCAGGTT	50	166
†7 (*HNF1* β gene)	‡/5’6-FAM/**TCAACACCTCCCAAGCACA**	‡**TGAGTCACAGCTGCCATGA**	50	197

According to DNA sequence (Ensembl: ENST00000375679); Bases that differ from the *CLCNKA* gene (Ensembl: ENST00000331433) are marked in bold; * primers kindly provided by the Hôpital Européen Georges Pompidou, Service de Génétique, Paris; † Control for the QMPSF technique. ‡ Previously designed primers [30].

In order to improve the genetic diagnostic the algorithm was as follows: first, detection of the previously described Spanish founder mutation (c.610G>A; p.Ala204Thr) [18]; second, detecting large deletions or duplications by MLPA and QMPSF; and third, sequencing of the coding and flanking regions of the whole *CLCNKB* gene.

### Statistics

Reported data correspond to the mean ± SD unless otherwise specified.

## Results

### Clinical results

Clinical symptoms consisted of polyuria, polydipsia, vomiting, constipation, salt craving, dehydration, hypotonia, and failure to thrive. In 8 cases (30,7%) a history of hydramnios was also recorded. Characteristically, all patients had hypokalemia (K^+^ 2,4 ± 0,5 mEq/l) of renal origin (FEK mean 32,9%), commonly associated with hypochloremia (Cl^-^ 91 ± 11 mEq/l), and metabolic alkalosis (plasma pH 7,48 ± 0,08, bicarbonate 30 ± 5 mEq/l). Increased plasma renin activity (mean 59,7 ng/ml/h), and aldosterone levels (mean 1020 pg/ml) were observed despite normal blood pressure, a marker of BS. At the time of the study two cases (7,7%) presented hypomagnesemia, and 5 (19%) nephrocalcinosis. Further, growth retardation and poor weight gain (mean Z-height -1,8, and Z-weight -2,7) were very common in the study cohort (Table 1).

### Mutation analysis in the *CLCNKB* gene

#### Spanish founder mutation detection (c. 610G>A; p.Ala204Thr)

We found that 23 patients (88%), belonging to 20 families (Table 4), presented with the Spanish founder mutation c.610G>A; p.Ala204Thr (exon 7) [18]. From those, 15 patients (15 families) were homozygous for the mutation (58%). Another 8 patients (5 families) were compound heterozygous (31%). We observed a high frequency (38/52 of the alleles) of the p.Ala204Thr founder mutation in our population.

**Table 4:** Molecular results for the *CLCNKB* gene in the studied cohort.

Patient	Sex	Ethnic origin	Exon	Mutation‡ 1	Mutation‡ 2	Father‡	Mother‡
SOR003	F	Spain	7	c.610G>A p.Ala204Thr		c.610G>A p.Ala204Thr	c.610G>A p.Ala204Thr
SOR005	F	Spain	7	c.610G>A p.Ala204Thr		c.610G>A p.Ala204Thr	c.610G>A p.Ala204Thr
SOR008	F	Spain	7	c.610G>A p.Ala204Thr		c.610G>A p.Ala204Thr	c.610G>A p.Ala204Thr
SOR009	F	Spain	7	c.610G>A p.Ala204Thr		c.610G>A p.Ala204Thr	c.610G>A p.Ala204Thr
SOR023	M	Spain	7	c.610G>A p.Ala204Thr		c.610G>A p.Ala204Thr	c.610G>A p.Ala204Thr
SOR025	F	Spain	7	c.610G>A p.Ala204Thr		c.610G>A p.Ala204Thr	c.610G>A p.Ala204Thr
SOR026	F	Spain	7	c.610G>A p.Ala204Thr		c.610G>A p.Ala204Thr	c.610G>A p.Ala204Thr
SOR039	F	Spain	7	c.610G>A p.Ala204Thr		c.610G>A p.Ala204Thr	c.610G>A p.Ala204Thr
SOR045	M	Spain	7	c.610G>A p.Ala204Thr		c.610G>A p.Ala204Thr	c.610G>A p.Ala204Thr
SOR047	M	Spain	7	c.610G>A p.Ala204Thr		c.610G>A p.Ala204Thr	c.610G>A p.Ala204Thr
SOR048	F	Spain	7	c.610G>A p.Ala204Thr		c.610G>A p.Ala204Thr	c.610G>A p.Ala204Thr
SOR050	M	Spain	7	c.610G>A p.Ala204Thr		c.610G>A p.Ala204Thr	c.610G>A p.Ala204Thr
SOR051	M	Spain	7	c.610G>A p.Ala204Thr		c.610G>A p.Ala204Thr	c.610G>A p.Ala204Thr
SOR062	M	Spain	7	c.610G>A p.Ala204Thr		c.610G>A p.Ala204Thr	c.610G>A p.Ala204Thr
SOR073	F	Spain	7	c.610G>A p.Ala204Thr			
SOR011	F	Spain	7 1-20	c.610G>A p.Ala204Thr	c. -19-? _2053+?del	c.610G>A p.Ala204Thr	c. -19-? _2053+?del
SOR024*	F	Spain	7 1-20	c.610G>A p.Ala204Thr	c. -19-? _2053+?del	c. -19-? _2053+?del	c.610G>A p.Ala204Thr
SOR024*	F	Spain	7 1-20	c.610G>A p.Ala204Thr	c. -19-? _2053+?del	c. -19-? _2053+?del	c.610G>A p.Ala204Thr
SOR054*	F	Spain	7 14	c.610G>A p.Ala204Thr	**c.1325A>G† p.Glu442Gly**	c.610G>A p.Ala204Thr	**c.1325A>G† p.Glu442Gly**
SOR054*	F	Spain	7 14	c.610G>A p.Ala204Thr	**c.1325A>G† p.Glu442Gly**	c.610G>A p.Ala204Thr	**c.1325A>G† p.Glu442Gly**
SOR054*	F	Spain	7 14	c.610G>A p.Ala204Thr	**c.1325A>G† p.Glu442Gly**	c.610G>A p.Ala204Thr	**c.1325A>G† p.Glu442Gly**
SOR057	F	Spain	7 14	c.610G>A p.Ala204Thr	**c.1325A>G† p.Glu442Gly**	**c.1325A>G† p.Glu442Gly**	c.610G>A p.Ala204Thr
SOR064	M	Spain	6 7	c.610G>A p.Ala204Thr	c.508G>A p.Val170Met	c.610G>A p.Ala204Thr	c.508G>A p.Val170Met
SOR063	M	Spain African	12 1-20	**c.1192_1203del12† p.Ile398_Thr401del**	c. -19-? _2053+?del	**c.1192_1203del12† p.Ile398_Thr401del**	c. -19-? _2053+?del
SOR076	F	Latin America	7 16	c.629C>T p.Ala210Val	**c.1756+1G>A† Splice defect**		
SOR021	F	Spain					

* Siblings

† These mutations (also marked in bold) have not been reported to date

‡ Numbering is according to DNA sequence (Ensembl: ENST00000375679)

(All patients were homozygous unless a second mutation is given; all parents were heterozygous for the given mutation)

#### MLPA and QMPSF analysis

**Figure 1:**
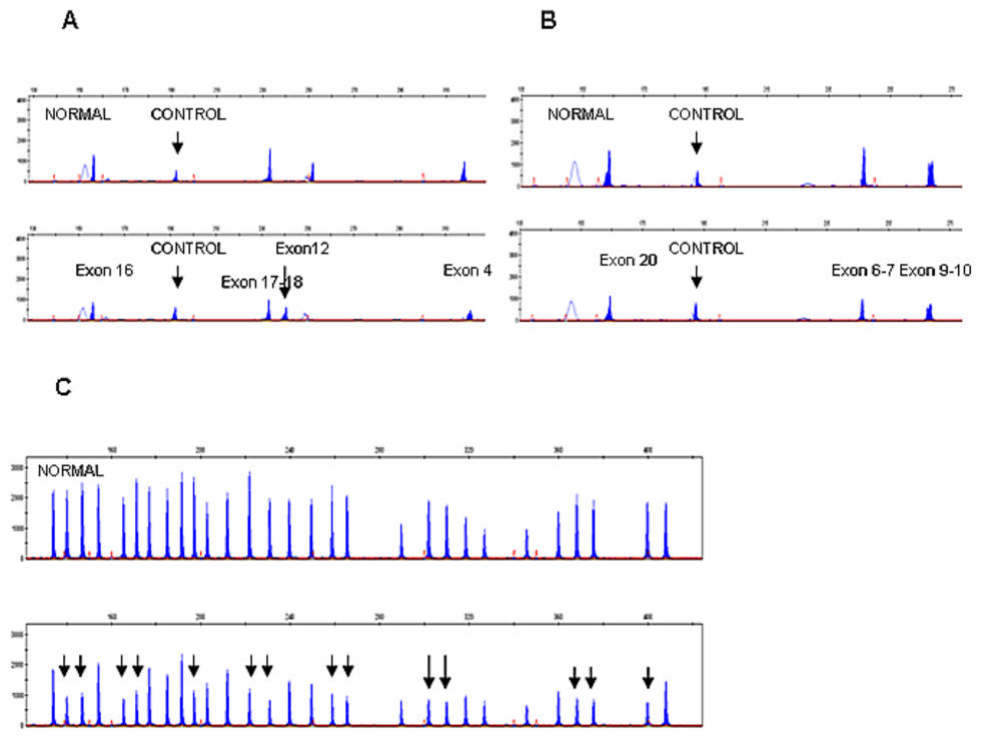
Detection of *CLCNKB* deletions by the QMPSF and MLPA techniques. QMPSF and MLPA electropherograms for the *CLCNKB* gene from controls (upper panels) and patients (lower panels). (A) QMPSF half doses for exons 16, 17-18, 12 and 4. The arrow shows the peak for exon 12 with the small deletion c.1192_1203del12 (patient SOR0063). Exon 7 of the *HNF1B* gene is the internal control. (B) QMPSF half doses for exons 20, 6-7 and 9-10. (C) For MLPA, each peak represents the exons for the *CLCNKB* gene and 15 control probes. The arrows show half doses for all the *CLCNKB* exons (patient SOR0011, family SOR0024 and SOR0063).

Among those 8 compound heterozygous patients for the founder mutation, MLPA and QMPSF techniques demonstrated that 3 patients (SOR011 and the 2 siblings of SOR024) had an entire gene deletion in one allele (c. -19-?_2053+? del). Further, we found another patient (SOR063) that had an entire gene deletion in one allele in combination with a small deletion of 12 base pairs (c.1192_1203del12) in the other, detected by direct sequencing. This small deletion is confirmed using the QMPSF technique; we observed an amplified fragment 12 base pairs smaller than the one corresponding to exon 12 (Figure 1). In all patients with the whole *CLCNKB* deletion, we confirmed that the probes for the nearly *CLCNKA*, *CASP9* and *PRDM2* genes included in the MLPA were present.

#### *CLCNKB* gene analysis

**Figure 2:**
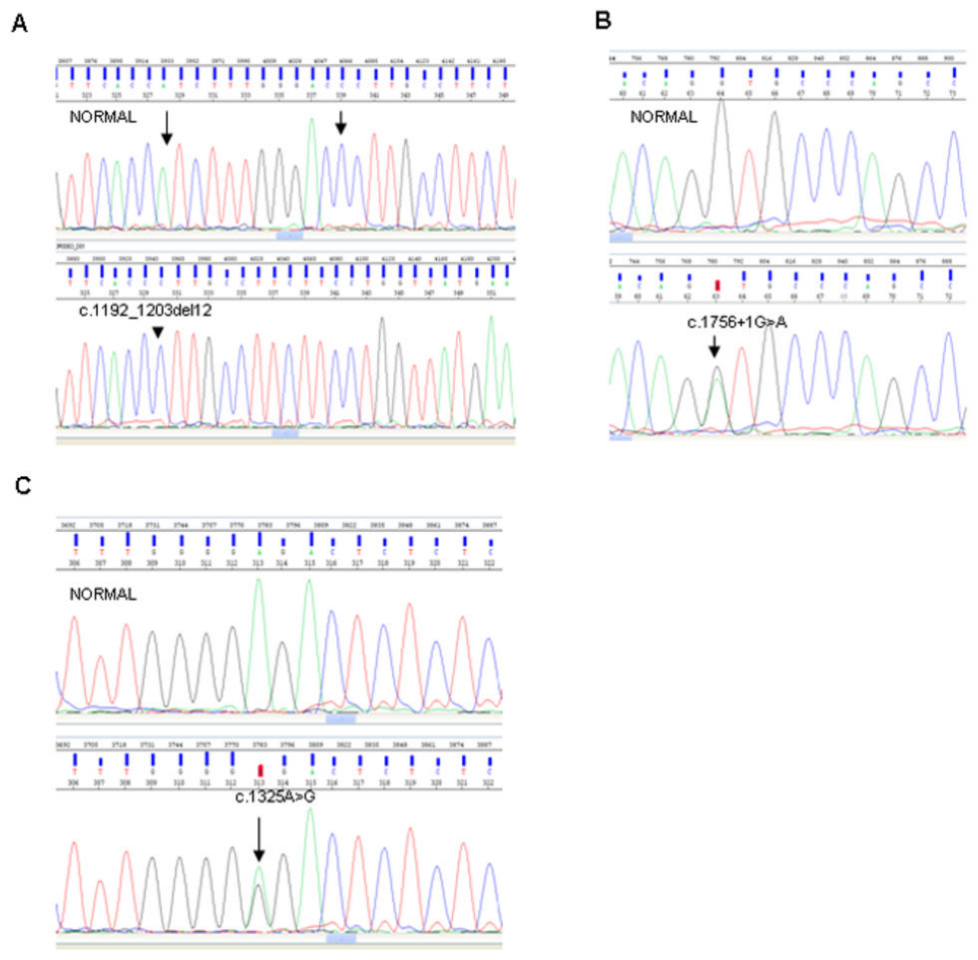
Detection of novel *CLCNKB* mutations by direct sequencing. Figures represent the sequencing chromatograms from controls (upper panels) and patients (lower panels). (A) Heterozygous c.1192_1203del12 mutation in exon 12; arrows show the extension of the deletion. (B) Heterozygous c.1756+1G>A mutation at the splicing site in exon 16. (C) Heterozygous missense mutation c.1325A>G in exon 14.

Complete structural *CLCNKB* gene analysis found missense mutations in the remaining 5 patients with the founder mutation: one case (SOR064) carried the described mutation c.508G>A; p.Val170Met (exon 6) [23], and 4 cases (SOR057 and the 3 siblings of SOR054) carried the novel mutation c.1325A>G; p.Glu442Gly (exon 14) (Figure 2).

Furthermore, we found two patients having other mutations in the *CLCNKB* gene. We observed one patient (SOR063) with a novel small deletion of 12 base pairs (c.1192_1203del12; p.Ile398_Thr401del) (Figure 2) in combination with the entire *CLCNKB* gene deletion (altogether, we found that 15% of cases -4/26- carried an entire gene heterozygous deletion within the cohort). The second patient (SOR076) presented the described mutation c.629C>T; p.Ala210Val (exon 7) [22] in combination with a novel splice-donor mutation c.1756+1G>A, a substitution of the 5´-donor splice site of intron 16 (Figure 2). Finally, in one case (SOR021), the molecular analysis of *CLCNKB* turned out to be negative.

We also detected 51 healthy relatives with heterozygous mutations in *CLCNKB* (43 presented the founder mutation p.Ala204Thr in heterozygosis), who were thus considered carriers in our genetic screening.

We tested 95 control subjects of Spanish ancestry in order to additionally confirm the pathogenicity of the novel mutations found in this study. None presented any of the mutations in either allele.

The complete description of the genetic results in our series is detailed in Table 4.

## Discussion

In the present study, we demonstrated that the Spanish p.Ala204Thr mutation, despite being very prevalent, is not the unique cause of type III BS in Spain, with other distinct *CLCNKB* gene mutations being observed. Importantly, the complete genetic study revealed three not previously reported *CLCNKB* mutations: p.Glu442Gly, c.1756+1G>A and p.Ile398_Thr401del.

We identified the genetic defect in almost all cases by polymerase chain reaction (PCR) and direct *CLCNKB* gene sequencing. Most of our cohort of BS patients presented the substitution of the hydrophobic non-polar amino acid alanine to the polar amino acid threonine at codon 204 in the fifth transmembrane domain, thus confirming our previous report regarding a founder mutation in most type III Bartter patients among Spanish population [18]. It has also been described that point mutations in the fifth transmembrane domain of the protein are associated with altered channel activity [24]. In previous studies realized by our group, 300 control subjects of Spanish ancestry were sequenced, none was homozygous for the p.Ala204Thr allele and it was identified in only 2 of 600 control chromosomes [18]. Therefore, that amino acid change represents a true loss of function mutation and not a common polymorphism.

It has been described that homozygous deletion encompassing the whole *CLCNKB* gene represents the most common molecular finding in type III BS [9]. Accordingly, we decided to screen for large rearrangements at the *CLCNKB* gene. We emphasize that in our study we have only found heterozygous deletions for the entire gene. *CLCNKA* and *CLCNKB* genes are separated only by 9,727 kb of genomic sequence, sharing a 94% of sequence identity and, therefore, it has been stated that there is a predisposition to a high rate of rearrangements by unequal crossing over [13], which would explain the high frequency of the whole gene deletion. Thus, some authors have suggested a complete gene analysis also in cases of an apparently homozygous mutation, due to the high possibility of being indeed hemizygous [25].

Among heterozygous cases with the founder mutation, we found two different mutations: the previously described mutation c.508G>A; p.Val170Met [23] and the novel mutation c.1325A>G; p.Glu442Gly. In the first one, the change of a hydrophobic valine to a nonpolar methionine (with a thioether group) causes a defective protein. In the second case, the exchange of a negative charged hydrophilic glutamic acid to a small uncharged glycine predicts a disruption of the charge distribution of the domain and the alteration of the conformation of ClC-Kb.

Other mutations were found in compound heterozygosis: one patient (SOR063) presented a not previously described small deletion of four amino acids in the paternal allele (c.1192_1203del12; p.Ile398_Thr401del) in combination with the entire gene deletion in the maternal allele. Lack of these four amino acids might lead to a defective chloride channel. The program Mutation t@sting predicts loss of one transmembrane helical domain and additional activations of splice sites; another patient (SOR076) presented the described mutation c.629C>T; p.Ala210Val [22]. Alanine 210 is a residue located within the transmembrane G helix. Mutations in many residues within the G helix have been described to cause functional alterations of chloride channels [26-28]. This mutation is present in combination with a novel heterozygous splice-donor mutation c.1756+1G>A. This guanine to adenine substitution might lead to the exon skipping and generation of an aberrant chloride channel ClC-Kb.

One case turned out to be negative in our genetic screening. Despite we cannot discard the possible presence of a deep intronic mutation or any complex rearrangement (i.e., intragenic inversions) not detected with our strategy, it is more plausible that this patient might have mutations in other transporter-codifying genes (*KCNJ1*, *SLC12A1*), or in the *SLC12A3* gene (OMIM *600968). Indeed, *SLC12A3* encodes for the thiazide-sensitive Na^+^-Cl^-^ cotransporter localized in the distal tubule. Mutations in this gene cause Gitelman syndrome [29], which is characterized by hypokalaemic metabolic alkalosis in combination with hypomagnesemia and hypocalciuria and may cause phenotypes that overlap with BS [17].

In conclusion, based on our results, we propose this algorithm as a way to reduce costs and accelerate the genetic study of the type III BS in the Spanish population: because the p.Ala204Thr mutation in *CLCNKB* gene is the most prevalent mutation in Spain, we propose to first look for this mutation in exon 7. Nevertheless, we have also found other mutations, including gross deletions. Therefore, should the p.Ala204Thr mutation not be detected in clinically diagnosed type III BS patients, whole gene sequencing, MLPA and QMPSF analyses must be carried out.
